# PDMS Microlenses for Focusing Light in Narrow Band Imaging Diagnostics

**DOI:** 10.3390/s19051057

**Published:** 2019-03-01

**Authors:** Adriana C. Costa, Sara Pimenta, João F. Ribeiro, Manuel F. Silva, Reinoud F. Wolffenbuttel, Tao Dong, Zhaochu Yang, José H. Correia

**Affiliations:** 1 Chongqing Key Laboratory of Colleges and Universities on Micro-Nano Systems Technology and Smart Transducing, Chongqing Engineering Laboratory for Detection, Institute of Applied Micro-Nano Science and Technology—IAMNST, Control and Integrated System, National Research Base of Intelligent Manufacturing Service, Chongqing Technology and Business University, Nan’an District, Chongqing 400067, China; adriana.c.costa@dei.uminho.pt (A.C.C.); Tao.Dong@usn.no (T.D.); Zhaochu.Yang@usn.no (Z.Y.); higino.correia@dei.uminho.pt (J.H.C.); 2 CMEMS-UMinho, Department of Industrial Electronics, University of Minho, 4800-058 Guimaraes, Portugal; jribeiro@dei.uminho.pt (J.F.R.); fsilva@dei.uminho.pt (M.F.S.); 3 Faculty of EEMCS, TUDelft, Mekelweg 4, 2628 CD Delft, The Netherlands; r.f.wolffenbuttel@tudelft.nl; 4 Faculty of Technology, Institute for Microsystems-IMS, Natural Sciences and Maritime Sciences, University of South-Eastern Norway (USN), Postboks 235, 3603 Kongsberg, Norway

**Keywords:** Narrow Band Imaging (NBI), Polydimethylsiloxane (PDMS) µ-lens, minimally invasive medical devices

## Abstract

Minimally invasive medical devices can greatly benefit from Narrow Band Imaging (NBI) diagnostic capabilities, as different wavelengths allow penetration of distinct layers of the gastrointestinal tract mucosa, improving diagnostic accuracy and targeting different pathologies. An important performance parameter is the light intensity at a given power consumption of the medical device. A method to increase the illumination intensity in the NBI diagnostic technique was developed and applied to minimally invasive medical devices (e.g., endoscopic capsules), without increasing the size and power consumption of such instruments. Endoscopic capsules are generally equipped with light-emitting diodes (LEDs) operating in the RGB (red, green, and blue) visible light spectrum. A polydimethylsiloxane (PDMS) µ-lens was designed for a maximum light intensity at the target area of interest when placed on top of the LEDs. The PDMS µ-lens was fabricated using a low-cost hanging droplet method. Experiments reveal an increased illumination intensity by a factor of 1.21 for both the blue and green LEDs and 1.18 for the red LED. These promising results can increase the resolution of NBI in endoscopic capsules, which can contribute to early gastric lesions diagnosis.

## 1. Introduction

Narrow Band Imaging (NBI) is an endoscopic diagnostic technique that uses narrow band filters at red, green, and blue (RGB) wavelengths [1,2]. The NBI technique presents a diagnosis improvement over the common white light, because the use of three wavelengths allows the capturing of images at different tissue depths. The blue light (at 415 nm) reproduces superficial images of the mucosa and capillaries, and corresponds to the main absorption peak of hemoglobin, which provides a good contrast of small vessels. The green light (at 540 nm) allows a deeper penetration into the tissue compared to blue light, and provides an intermediate image. Finally, the red light (at 600 nm) allows the deepest penetration, corresponding to the imaging of deeper vessels and allowing the analysis of the submucosa [1,2]. The light penetration range into the tissue varies from 0.15 to 0.30 mm with the NBI technique [2].

The diagnosis of some diseases, such as Barret’s esophagus and gastric cancer, has become faster and more accurate with the NBI technique [1,2]. Increasing the luminous intensity at the target area of interest is an important requirement for this imaging diagnostic method, since it allows obtainment of a higher-quality image.

Endoscopic imaging uses light not only to obtain images of internal tissue, but also for therapeutic applications [3,4]. Miniaturization and high sensitivity are increasingly becoming key challenges for imaging apparatus, not only to minimize patient discomfort but also to obtain a correct diagnosis [5]. Over the last few years, the advances in endoscopic imaging have provided higher-quality images and allowed reaching more challenging places, such as the small intestine [6]. Endoscopic images acquired using the NBI technique aim at identifying morphological changes in the micro-vessels’ density in the early-stages pathological neoplasm tissue. Typically, white light is used for illumination. However, some lesions in an early stage are located at deep regions of the tissue and are relatively asymptomatic. The implementation of NBI in minimally invasive medical devices allows an early diagnosis [5,7].

An endoscopic capsule is a minimally invasive medical device that provides a faster diagnosis with less pain and discomfort to the patient [4,8]. The first endoscopic capsule equipped with complementary metal-oxide semiconductor (CMOS) image technology was described by Qureshi [8]. Since then, several companies have produced endoscopic capsules that revealed highly efficient results in reducing gastrointestinal bleeding detection [9,10]. Common to all commercial capsules is the transparent dome in the end of light emitting diodes (LEDs) and the CMOS imager, which places the inspected tissue at approximately 10 mm from the light source. LED sources are mainly characterized by their relatively small dimensions and their low power consumption, which make them ideal for minimally invasive medical devices. The wide viewing angle (120–130°) that also characterizes LEDs results in a loss of light intensity at the targeted area of interest. Therefore, the development of a system capable of increasing the light intensity at the target area of interest is a crucial requirement.

This work presents a µ-lens placed on top of LEDs to increase the light intensity at the target area of interest, without increasing the power consumption of the system. The µ-lenses are used in many optical applications, such as optical communications, optical systems for digital imaging, biomedical optical imaging, and optoelectronics [11,12,13,14,15]. There are several reported methods to produce µ-lenses, including dielectrophoresis force [16], lithography [17], ink jet printing [18], RIE (reactive ion etching) [19], the Litographie, Galvanoformung, and Abformung (LIGA) process [20], hot embossing [21], deep proton irradiation [22], and nanoimprint techniques [23]. Most of these techniques produce µ-lenses using a mold and require specific facilities in the lab. In this paper, a hanging droplet method was used to produce the polydimethylsiloxane (PDMS) µ-lens. The hanging droplet method is a very simple and low-cost process with excellent performance that does not require complex manufacturing equipment [24].

In comparison with other manufacturing methods, the hanging droplet method does not require a mold, and therefore, does not present mechanical defects resulting from the mold injection and stamping processes. A similar method was described by Grimaldi et al. [25], using Polymethylmethacrylate (PMMA) to produce an array of µ-lenses, applying thermal pulses to the reservoir drop in a fixed position parallel to the target substrate. Contrary to this method, fabrication of a PDMS µ-lens by the hanging droplet method does not require temperature processes and allows a control on the µ-lens specifications (thickness, focal point, etc.) due to the volume of the PDMS solution [26]. The PDMS biocompatible polymer was chosen due to the following characteristics [26,27]: (1) strong adhesion and internal cohesion; (2) good optical properties, i.e., a refractive index of approximately 1.4 (below 1600 nm) [28]; (3) optical transparency in the visible region (>95%); and (4) Young’s modulus of 1 MPa. The flexibility and transparency of this polymer also enables its use as a micro-electro-mechanical systems (MEMS) substrate or for microfluidic devices [29,30,31]. There are also some research about arrays of PDMS µ-lenses, proving the reproducibly of this material to replace the most conventional glass-based µ-lenses [32,33].

The authors have also reported hanging droplet method to fabricate PDMS µ-lenses for biopsy microsystems, achieving an image magnification by a factor 4 and a 30% improvement in optical irradiance from LED illumination. The focal point was 3.15 mm, with a numerical aperture (NA) of 0.29 and depth of focus (*δz*) of 5.76 µm [26].

Several authors have developed lens designs for optimization of the endoscopic capsule performance, ranging from the development of wide-angle lenses for enhanced imaging quality [34], to the development of a hybrid lens design for a better image of the gastrointestinal tract (dual view) [35].

**Figure 1:**
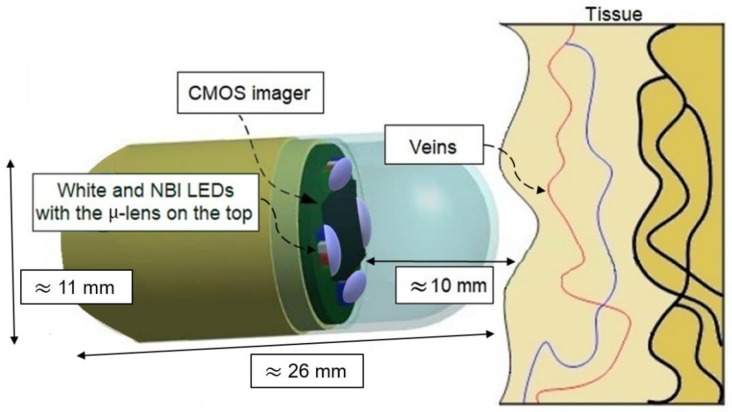
Artwork of an endoscopic capsule with Narrow Band Imaging (NBI) light emitting diodes (LEDs) and the polydimethylsiloxane (PDMS) u-lenses on their tops. CMOS: complementary metal-oxide semiconductor.

In this work, the goal is to produce a PDMS µ-lens to be used on the top of RGB LEDs, with a focal length of ≈10 mm, to increase the light intensity at a target area of interest without increasing the power consumption of the medical device (Figure 1). The objective is to improve the resolution of the NBI technique in endoscopic capsules. Improvements on NBI endoscopic capsules contributes to earlier diagnosis in several types of gastrointestinal tract tumors. Commercially available LEDs in the RGB spectrum were used and the fabricated PDMS µ-lenses were placed on their tops. The combined device was subjected to measurements to validate the light intensity improvement at different wavelengths.

## 2. Design and Simulations

### 2.1. Microlens Design

The two main physical characteristics of a µ-lens are the central height and the base diameter, which define the radius of curvature and consequently the focal point. The µ-lens curvature radius (R) is calculated according to Equation (1), where h is the central height and d is the base diameter of the µ-lens given by:

(1) $$R=\frac{h}{2}+\frac{d^{2}}{8h}.$$

The focal point (f) of the µ-lens, for thin lens approximation [36], is given by Equation (2):

(2) $$\frac{1}{f}=\frac{n_{1}-n_{2}}{n_{1}}\left(\frac{1}{R_{1}}-\frac{1}{R_{2}}\right),$$

where $n_{1}$ and $R_{1}$ are the refractive index and the curvature radius of the first surface, respectively, and $n_{2}$ and $R_{2}$ are the refractive index and the curvature radius of the second surface, respectively. The first medium is air ($n_{1}=1$) and the second medium is PDMS ($n_{2}\approx 1.4$) [28]. Considering a plan-convex µ-lens, $R_{1}\to \infty$. Therefore, the focal point is obtained by Equation (3):

(3) $$f=\frac{4h^{2}+d^{2}}{8h\left(n_{2}-1\right)}.$$

**Figure 2:**
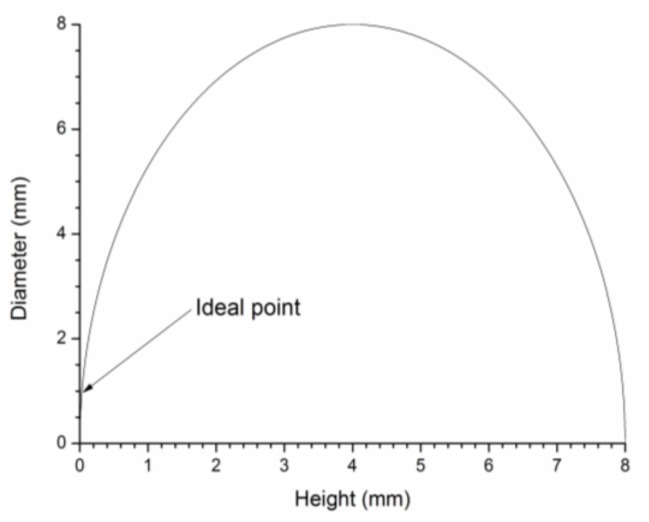
Graphical representation of Equation (3) considering a PDMS µ-lens with a focal point of 10 mm. The ideal base diameter and central height of the µ-lens are indicated.

Considering the application in medical instruments, such as the endoscopic capsule, a focal point of 10 mm is necessary to increase the light intensity in the area of interest (gastrointestinal tissue). Figure 2 presents a graphical representation of Equation (3), considering a PDMS µ-lens with a focal point of 10 mm. To ensure a 10 mm focal length, the PDMS µ-lens central height and base diameter need to correspond to a point on the line of the semi-circle traced in Figure 2. In order to ensure the minimal dimensions of the µ-lens and the minimal volume of PDMS used, the ideal point is the one closest to the origin (as indicated in Figure 2).

### 2.2. Microlens Optical Simulation

The ZEMAX^®^ optical design software (version 2009) was used to simulate the µ-lens on top of each commercially available LED with three different wavelengths. The LEDs used were Vishay ChipLED 0402: blue (470 nm), yellow green (573 nm), and super red (631 nm). These specific LEDs were chosen because of: their dimensions, suitable for integration on an endoscopic capsule; their specific wavelength, fitting the NBI technique; and their geometry, which is appropriate for the fabrication of the µ-lens in a planar surface. The non-sequential simulation mode included in the ZEMAX^®^ software was selected for observing the dispersed rays. In order to design the µ-lens, the PDMS material properties were first inserted in the glass catalogue, based on the Sellmeier equation expressed in [36], Equation (4):

(4) $$n^{2}=1+\frac{-2.30744\times \lambda^{2}}{\lambda^{2}+0.02605}+\frac{1.85952\times \lambda^{2}}{\lambda^{2}+0.01362}+\frac{1.39944\times \lambda^{2}}{\lambda^{2}+0.01349},$$

where n represents the refractive index and λ the wavelength. According to Equation (4), the PDMS refractive index varies from approximately 1.422 to 1.398 in the wavelength range between 0.4 and 1.8 µm, respectively.

For the optical design, the parameters of the plan-convex µ-lens and its substrate were first introduced in the sequential mode in the ZEMAX^®^ software, as represented in Table 1. Each µ-lens has 3.9 mm of base diameter and 0.52 mm of central height. The substrate was a thin cover glass approximately 0.1 mm thick (Marienfeld No.0, 0100032). Then, these components were converted into a single non-sequential mode in the ZEMAX^®^ software. Through this mixed mode ray tracing, the scattered rays were considered for the final result. The number of rays in the non-sequential mode was tested in a cycle of simulations until having a variation of the final result lower than 5%. This value was fixed in 10^6^ analysis rays. Moreover, in order to establish an optical design according to real components, the LEDs were represented by a radial source. The parameters, such as active area, wavelength, power intensity, and relative intensity variation due to angle, were inserted in the ZEMAX^®^ software according to the datasheet of each LED. Table 2 shows the ZEMAX^®^ parameters used for the light sources (LEDs). A rectangular detector with an absorber surface was selected with the dimensions of the photodiode used in the experimental setup, which presents an active area of 13 mm^2^ (Si calibrated photodiode, model FDS 100-CAL from Thorlabs). This photodiode was selected because of its range (350–1100 nm), responsivity, and also the dimensions of the light sources (LEDs).

**Figure 3:**
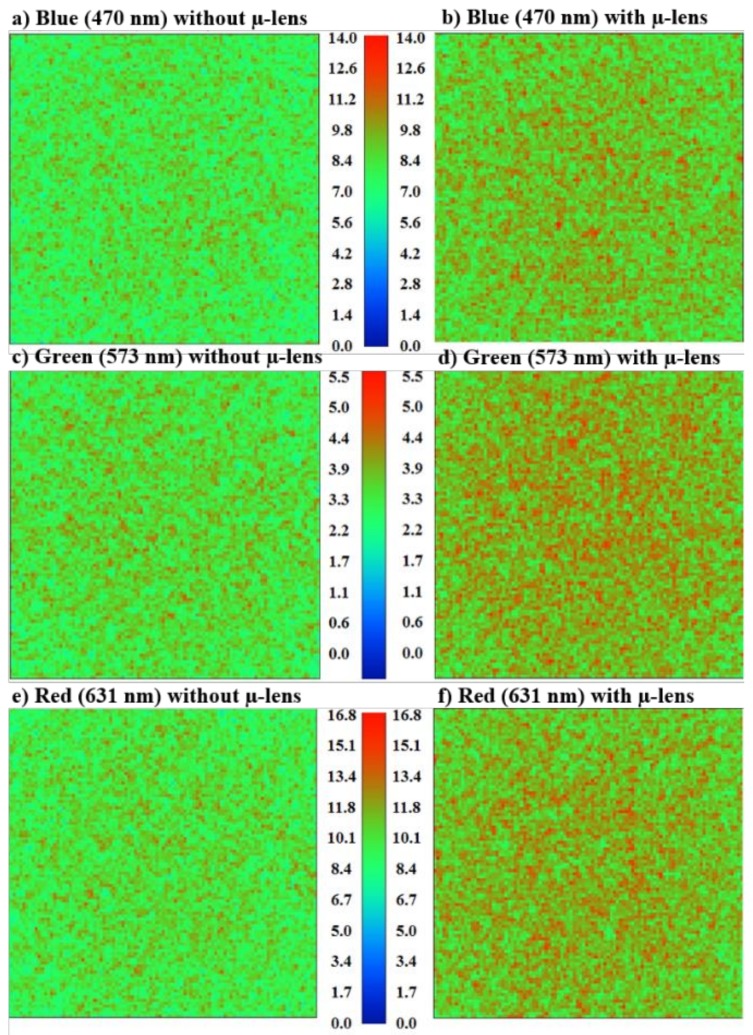
ZEMAX^®^ irradiance maps simulation results for: blue LED (**a**) without µ-lens and (**b**) with µ-lens; green LED (**c**) without µ-lens and (**d**) with µ-lens; and red LED (**e**) without µ-lens and (**f**) with µ-lens.

Figure 3 presents the irradiance maps for all the LEDs, with and without µ-lens, plus the substrate. The blue LED has a peak irradiance power of 11.75 W/m^2^ without µ-lens and 13.98 W/m^2^ with µ-lens, which represents an increase by a factor of 1.19 using the µ-lens. The green LED has an irradiance power of 4.70 W/m^2^ without µ-lens and 5.43 W/m^2^ with µ-lens, which represents an increase by a factor of 1.16 using the µ-lens. The red LED has an irradiance power of 14.23 W/m^2^ without µ-lens and 16.78 W/m^2^ with µ-lens, which represents an increase by a factor of 1.18 using the µ-lens.

## 3. Microlens Fabrication

**Figure 4:**
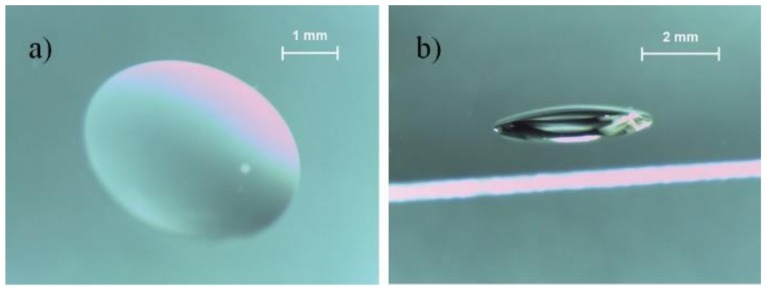
Photographs of the µ-lens produced, obtained with a Leica M80TM stereo microscope: (**a**) top view and (**b**) side view.

The PDMS from Dow Corning Sylgard^®^ 184 solution is obtained by mixing the PDMS base and the curing agent, with a 10:1 ratio. The resulting solution of the base and curing agent inevitably presented residual bubbles, which were removed using a vacuum chamber. Subsequently, the PDMS was pipetted to a thin cover glass, used as substrate. Then, the substrate was positioned upside down inside an oven at 70 °C for 30 min for curing. With the gravitational interference exercised over the entire sample, the PDMS forms a drop. This method is generally referred to as hanging droplet and does not require any special equipment, resulting in a simple and low-cost method for fabricating µ-lenses. For the required 10 mm focal length, 0.8 µL of PDMS was pipetted on the top of the glass substrate, forming a µ-lens with 3.9 mm base diameter and 0.52 mm central height. An approximation to the ideal point shown in Figure 2 is very difficult, due to the low PDMS volume required, which compromises the reproducibility of the process. Figure 4 shows the resulting µ‑lens, captured with a model M80TM stereo microscope from Leica and processed in the Leica LAS™ software, version 3.4.0.

The thermal reflow technique, reported by Roy et al. [19], allowed the production of a µ-lens with a height of 11 µm and a focal point of 226.6 µm. This technique requires a higher spending on manufacturing machinery, particularly for application in endoscopic capsules where the focal point required is approximately 10 mm. Chen et al. reported on a microlens with a diameter between 600–1400 µm, a focal length from 3.82 to 10.64 mm, and a NA between 0.09 and 0.24 [37]. The work reported by Fuh et al. presents a lens with 10.2 mm focal length and 1.5 mm height, produced using the hanging droplet technique [38]. The µ-lens produced in this work features a focal length of approximately 10 mm through a lens that is 3 times smaller in height (0.52 mm).

## 4. Results and Discussion

### 4.1. Microlens Transmittance

**Figure 5:**
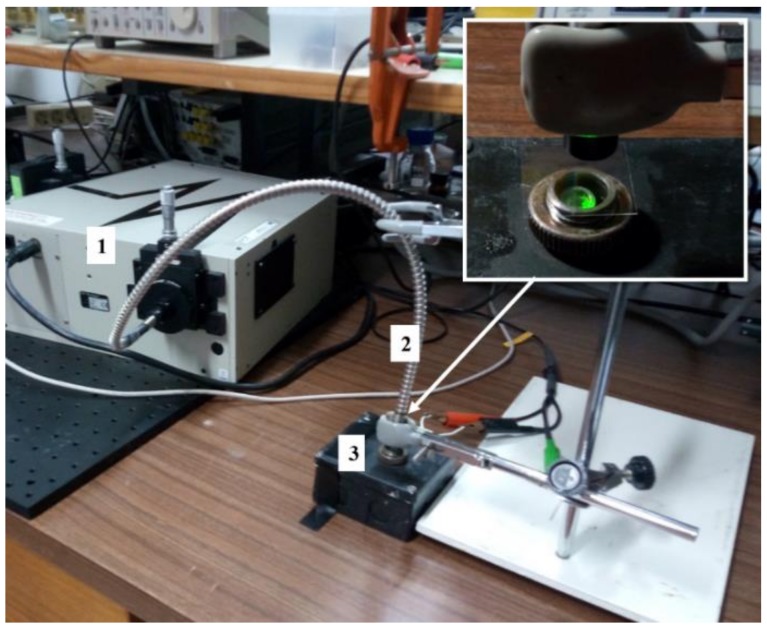
Optical setup created to measure the transmittance which includes: (**1**) monochromator (Newport 74125), (**2**) fused silica fiber-optic (Newport 77563), and (**3**) Si photodiode (Hamamatsu S1336-5BQ).

The transmittance was measured in order to quantify the optical losses inherent to the thin glass substrate and PDMS material. The optical setup used for transmittance measurements includes a monochromator model 74125 from Newport, a fused silica fiber-optic model 77563 (also from Newport), and a Si photodiode model S1336-5BQ from Hamamatsu (Figure 5). A wavelength range from 400 to 700 nm was chosen for measurements, since it covers the three LEDs used in this work.

**Figure 6:**
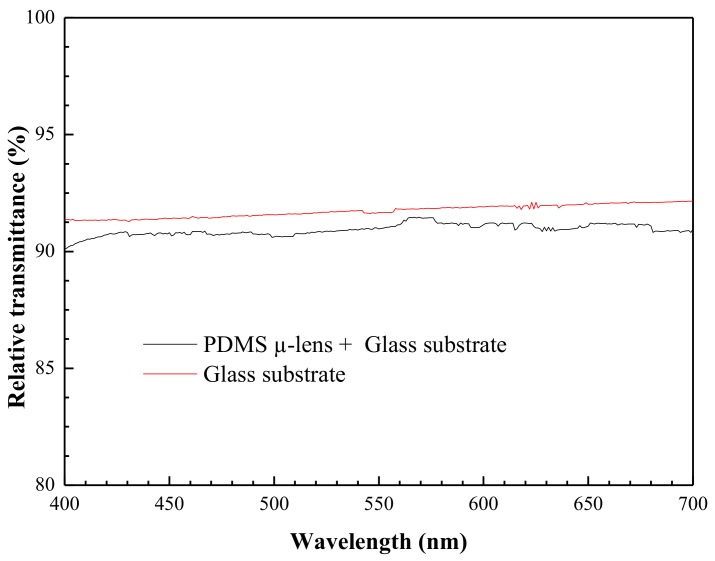
Relative transmittance of the glass substrate and the PDMS µ-lens plus the glass substrate for the 400 to 700 nm wavelength range.

The transmittance results are presented in Figure 6. The optical setup reference was obtained without anything between the light source and the photodetector. Then, the substrate or the µ-lens plus the substrate were placed in front of the light source for measurement. The transmittance of each sample was calculated by the ratio between the current intensity obtained with the sample and the obtained reference. The loss in light intensity by the µ-lens plus the substrate represented less than 10% for the 400 to 700 nm wavelength range and was mostly due to the thin glass substrate. The PDMS proved to be a good choice as the bulk material for the production of µ-lenses, since it has a transmittance of about 98% in the wavelength range considered.

### 4.2. Microlens Optical Characterization

The numerical aperture (NA) and the depth of focus (δz) are given by Equations (5) and (6), respectively [11]. Table 3 presents the calculated PDMS refractive index (n) by Equation (4), focal point (f) by Equation (3), and δz for each LED wavelength (λ), considering the fabricated µ-lens with a 3.9 mm base diameter and a 0.52 mm central height.

(5) $$NA=\frac{d}{2f},$$

(6) $$\delta z=\frac{\lambda }{NA^{2}}.$$

**Figure 7:**
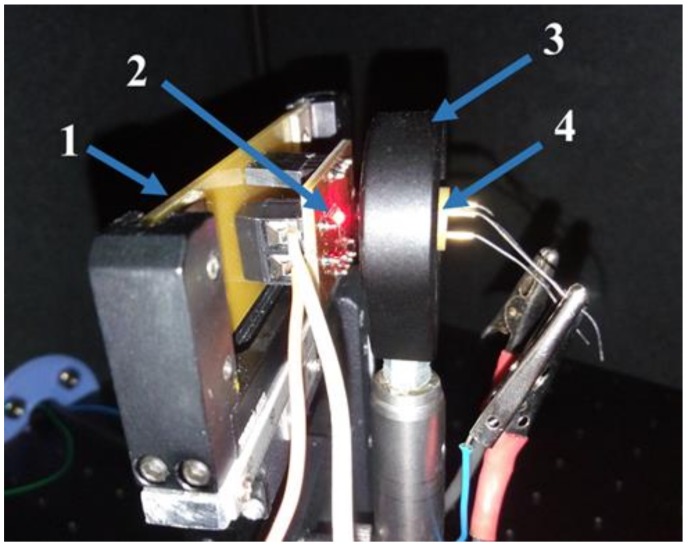
Optical setup for irradiance measurements. (**1**) LED holder, (**2**) LED, (**3**) photodiode holder, and (**4**) Thorlabs FDS 100-CAL Si calibrated photodiode.

The irradiance measurements were made to analyse the efficiency of the µ-lens on top of each LED, comparing with the optical simulations. An optical setup consisting of a photodiode, model FDS 100-CAL Si calibrated photodiode from Thorlabs with a 13 mm^2^ active area was used (the same used in the ZEMAX^®^ simulation). The photodiode has approximately a 10 mm distance from each LED, as required by the application, and was centred with each LED to ensure maximum irradiance measurements (Figure 7). For each LED, the irradiance values were recorded for the set-up with and without the µ-lens plus the substrate, and are presented in Table 3. The simulation results were also added to Table 3 for a faster comparison.

Table 3 presents the optical characterization and the irradiance values (simulation and experimental) of each wavelength with and without the µ-lens plus the substrate. The irradiance values achieved in the ZEMAX^®^ optical simulations were higher than with the experimental setup, but the increase factors are in the same range of the experimental results. The differences between the simulations and experimental results may be due to several reasons: the conditions assumed in the simulations may not be exactly the same as the actual conditions during the experimental measurements and the optical properties of the fabricated PDMS can be slightly deviated from those used in the simulations. More specifically, the limited validity of the following assumptions should be mentioned: (1) the simulation is based on a perfect alignment between the light sources and the detector; (2) in the simulation, the distance between the light source and the photodiode is precisely 10 mm; (3) the simulated µ-lens has a perfect surface profile, without roughness.

## 5. Conclusions

A PDMS based µ-lens was fabricated using a simple, low-cost, and low-temperature hanging droplet method. The µ-lens will be used on top of RGB LEDs dedicated to NBI technique. The result is a biocompatible µ-lens capable of increasing the light intensity at a target area of interest. Without increasing the power consumption or the dimensions of a medical device, the solution presented here can be applied on gastrointestinal noninvasive platforms, such as an endoscopic capsule with NBI diagnostic capabilities. Optical simulation, fabrication process, and characterization of the µ-lens were presented. The µ-lens has a 3.9 mm base diameter, 0.52 mm central height, and a focal length of approximately 10 mm, thus satisfying the requirements of the application. Experimentally, the light intensity was increased by a factor of 1.21 for both the blue and green LEDs and 1.18 for the red LED. Therefore, in an autonomous medical device, such as a NBI endoscopic capsule, where the power supply is a battery with limited time of use, the PDMS µ-lens will be a breakthrough for increasing illumination intensity without increasing the size of the device. Thus, NBI will become a technique with higher resolution for the early diagnosis of gastrointestinal lesions.

## Figures and Tables

**Table 1:** The µ-lens data in sequential mode, using the ZEMAX^®^ optical software. OBJ: object. STO: stopping. IMA: image. BK7: optical borosilicate glass.

Surface	Type	Comment	Radius (mm)	Thickness (mm)	Glass	Semi Diameter (mm)	Conic (mm)
OBJ	Standard	Optical dome	Infinity	Infinity	-	0.000	0.000
1	Standard	Substrate	Infinity	0.100	BK7	9.000	0.000
2	Standard	Lens	Infinity	0.520	PDMS	1.950	0.000
3	Standard	Lens	−3.916	0.520	-	1.950	−1.958
STO	Standard	Aperture stop	Infinity	0.000	-	0.000	0.000
IMA	Standard	Image sensor	Infinity	-	-	0.000	0.000

**Table 2:** LEDs data in non-sequential mode, using the ZEMAX^®^ optical software.

Object Type	Power (Watts)	X Half Width (mm)	Y Half Width (mm)	Minimum Angle (°)	Maximum Angle (°)	I(0); I(10); I(20); I(30); I(40)	I(50); I(60); I(70); I(80); I(90)	Wavelength (µm)
Source Radial	2.7 × 10^−3^	0.07	0.07	0	90	100; 100; 95; 90; 85	70; 50; 30; 15; 0	0.470
Source Radial	1 × 10^−3^	0.07	0.07	0	90	100; 100; 95; 90; 85	70; 50; 30; 15; 0	0.573
Source Radial	3.3 × 10^−3^	0.07	0.07	0	90	100; 100; 95; 90; 85	70; 50; 30; 15; 0	0.631

**Table 3:** Optical characterization and irradiance results of the ZEMAX^®^ optical simulation and experimental measurements for the blue, green, and red LEDs, with and without µ-lens. “Sim” are the simulation results and “Exp” are the experimental results.

Optical Analysis					
Wavelength (nm)			470	573	631
n			1.416	1.411	1.409
f (mm)			9.414	9.529	9.575
δz (mm)			0.011	0.014	0.015
Irradiance (W/m^2^)	Without µ-lens	Sim	11.75	4.70	14.23
		Exp	0.686	1.46	5.18
	With µ-lens	Sim	13.98	5.43	16.78
		Exp	0.832	1.76	6.12
	Increase factor	Sim	1.19	1.16	1.18
		Exp	1.21	1.21	1.18
